# Psychosocial impact of COVID-19 pandemic: experience of healthcare workers in Qatar

**DOI:** 10.3389/fpubh.2023.1283925

**Published:** 2023-10-19

**Authors:** Ahmad R. Al-Qudimat, Kalpana Singh, Emad Mustafa, Abdulqadir J. Nashwan, Raed M. Al-Zoubi, Aksam Yassin, Omar M. Aboumarzouk, Abdulla Al-Ansari

**Affiliations:** ^1^Surgical Research Section, Department of Surgery, Hamad Medical Corporation, Doha, Qatar; ^2^Department of Public Health, College of Health Sciences, QU-Health, Qatar University, Doha, Qatar; ^3^Department of Nursing Research, Hamad Medical Corporation, Doha, Qatar; ^4^Department of Biomedical Sciences, College of Health Sciences, QU-Health, Qatar University, Doha, Qatar; ^5^Department of Chemistry, College of Science, Jordan University of Science and Technology, Irbid, Jordan; ^6^Center of Medicine and Health Sciences, Dresden International University, Dresden, Germany; ^7^School of Medicine, Dentistry and Nursing, The University of Glasgow, Glasgow, United Kingdom

**Keywords:** COVID-19, healthcare, workers, psychosocial, impact, Qatar

## Abstract

**Background:**

The COVID-19 pandemic has had a profound and global impact on healthcare systems worldwide, presenting unprecedented challenges for healthcare workers (HCWs) on the front. We aimed to evaluate the prevalence of anxiety and depression symptoms during the coronavirus pandemic among healthcare professionals in Qatar.

**Methods:**

A cross-sectional study where an electronic questionnaire containing demographics, and psychosocial questions were made on Google Docs and Microsoft Team, and were sent through email and WhatsApp to healthcare workers, including doctors, nurses, allied health and others working at Hamad Medical Corporation in Qatar, from June 1, 2021, to January 1st 2023. ANOVA, *t*-test and multiple linear regression were used to see the association between the psychological factors and sociodemographic variables using STATA version 17 software.

**Results:**

A total of 829 participants were included in this study (response rate: 55%). The average age of the participants is 36.0 ± 7.1; 65.9% were males; 2.3% were doctors and 53% were nurses, 38.7% were allied healthcare professionals and 6% were others. Psychological, social effects, and workplace were shown to significantly related to their marital status, career, and hospital setting (*p* < 0.01 for each). Similar to this, dealing with COVID-19 patients and their education level with the length of time working at the designated facility were all connected with the health professional safety score (*p* < 0.05).

**Conclusion:**

During the COVID-19 epidemic, healthcare workers in Qatar experienced a high incidence of negative psychosocial symptoms. To alleviate these outcomes, it would be useful to implement screening procedures for such symptoms and to devise preventive measures accordingly.

## Graphical abstract

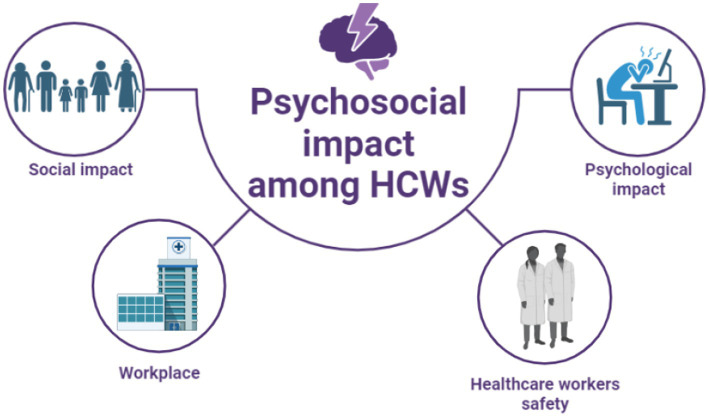

## 1. Introduction

The COVID-19 pandemic has had a profound and global impact on healthcare systems worldwide, presenting unprecedented challenges for healthcare workers (HCWs) on the front lines (1, 2). In response, various precautionary measures such as self-quarantine, social distancing, mandatory mask-wearing, and travel restrictions have been implemented to mitigate the spread of the highly contagious COVID-19 virus (3–8). However, these measures coupled with the suspension of elective medical procedures and strain on healthcare resources have significantly burdened HCWs and amplifying the pressure and presenting immense challenges (9–14).

Healthcare workers have faced not only an increased risk of infection but also prolonged periods of wearing cumbersome personal protective equipment (PPE), extended work shifts, and overwhelming patient caseloads (15–18). Furthermore, many studies reported the adverse effects of prolonged PPE usage. These effects include headaches, difficulty in breathing, and impaired cognition. Moreover, the continuous use of PPE interferes with vision, and communication, and disrupts thermal equilibrium (19–23). These demanding circumstances have taken a toll on the mental well-being of HCWs, leading to psychological distress, anxiety, sadness, and potential post-traumatic stress symptoms (24). Research conducted in countries such as the United States, Italy, and China has consistently reported high levels of anxiety, depression, and insomnia among HCWs during this unprecedented global health crisis (5, 25, 26).

On the other hand, several reports documented the diverse coping mechanisms adopted by healthcare workers. These strategies encompass seeking psychological support through counseling and therapy, engaging in stress-relieving activities such as physical exercise, meditation, and yoga, nurturing peer support from family and friends, as well as prioritizing effective self-care routines, and others. These endeavors played a critical role in preserving resilience and upholding an exceptional standard of patient care during this challenging period (27–30).

Despite the global recognition of the psychological impact on HCWs, limited research exists specifically examining the psychosocial effects of the COVID-19 pandemic on HCWs in the Gulf region, particularly in Qatar. To bridge this knowledge gap, the present study conducted a comprehensive cross-sectional examination of HCWs, encompassing various professional roles within significant hospitals under the Hamad Medical Corporation (HMC).

## 2. Methodology

### 2.1. Design

The study used descriptive, cross-sectional hospital-based study.

### 2.2. Setting and samples

The study included a total of 829 healthcare workers (HCWs), including doctors, nurses, pharmacists, laboratory technicians, ambulance staff, and administrative personnel working in four major hospitals under Hamad Medical Corporation (HMC) in Qatar between January 2021 and December 2022. The response rate for this study was 78% across various hospitals, including Hamad General Hospital and HMGH. Incomplete surveys from HCWs were excluded from the study. We utilized a convenience sampling method, and the sample size was determined using the following equation: *n* = [(*Z*_0.95_)^2^ × *p* × (1 − *p*)]/(0.05)^2^, where *n* represents the sample size, *Z*: constant (1.96), *p*: is the estimated proportion or prevalence that meets our criteria.” *p* will be set as 0.5, as the proportion is not known. To attain a confidence level of 95% with a precision of +/− 0.05, the recommended calculated sample size is a minimum of 500 participants.

Data were collected using an anonymous online questionnaire. The use of an online survey form was conducted on Google Docs and Microsoft Team forms in English and sent to healthcare workers via email and WhatsApp. There was no direct contact or face-to-face interaction with the HCWs.

### 2.3. Questionnaire

We developed a questionnaire using multiple English-language tools (31–33). The developed tools in consultation with mental health professionals. We conducted a pilot study involving 20 conveniently selected HWCs. We discussed with them the comprehensiveness, language, and grammar of the questions.

To assess the face and content validity of the questionnaire, we distributed it to four reviewers, consisting of two mental health professionals and two senior researchers. Each reviewer was asked to independently rate each item in the questionnaire and provide feedback on its readability, comprehensiveness, clarity, language, and grammar. Upon analyzing the results, we found that the questionnaire demonstrated accepted content validity.

### 2.4. Description of the data collection tool

The questionnaire consists of five sections, developed through an extensive literature review. The first section focuses on demographic characteristics and background information, such as age, sex, marital status, education level, nationality, specialty, hospital name, living status, family members, and other relevant details using multiple choice questions. The second section addresses 9 questions related to psychological impact, while the third section focuses on social impact with 5 questions. The fourth section delves into the workplace impact with 6 questions, and finally, the fifth section covers 4 questions on health professional safety. Likert scale (strongly disagree, disagree, neutral, agree, strongly agree) was used to answer questions for section 2 till section 5.

### 2.5. Statistical analysis

Descriptive statistics were calculated for the demographics and dependent variables of the study participants. Pearson correlations were used to examine the relationships between the four main variables, i.e., psychological impact, social impact, workplace and health professional safety. We used Q–Q plot and P–P plot and Schapiro Wilk test to check the normal distribution of psychological impact, social impact, workplace and health professional safety variables. ANOVA and *t*-tests were conducted to compare the level of psychological well-being, needs, resources, and job support satisfaction between the sector and socio-demographic characteristics (age, gender, education level, nationality, specialty, working with COVID-19 patient). A multiple linear regression was used to see the association between the psychological well-being, social impact, workplace and health professionals’ safety with the different sociodemographic variables. All statistical analysis was done using STATA 17 software with statistical significance level *p* < 0.05.

### 2.6. Ethical considerations

This study was conducted in accordance with the principles of the Declaration of Helsinki. Ethical approval for the study was obtained from HMC (Ethical Approval Number: MRC-01-21-235), and consent was obtained from all participants. Participants were provided with information about the study’s objectives, and assurance of the confidentiality of all shared information was given.

## 3. Results

### 3.1. Participant characteristics

The questionnaire was distributed via email and WhatsApp, and 829 people responded in total. The age of healthcare professionals was 36 ± 7.1. Moreover, 65.9% of the population were men, 70.8% were married, and 76% had a bachelor’s degree. Eighty-three percent (83.2%) of the participants were Asian, with 58.1% of them working at Hamad General Hospital and 35.6% at Hazm Mebaireek General Hospital (HMGH) (Table 1).

**Table 1:** Characteristic of HCWs.

Variables	Label	*N* = 829
Age		36.0 (7.1)
Sex	Female	282 (34.1%)
	Male	544 (65.9%)
Marital status	Married	587 (70.8%)
	Single	235 (28.3%)
	Widow/divorced	7 (0.8%)
Education level	Bachelor	627 (76.0%)
	Diploma	44 (5.3%)
	Master	115 (13.9%)
	Ph.D.	39 (4.7%)
Nationality	African	104 (14.5%)
	Asia	596 (83.2%)
	Europe	6 (0.8%)
	North America	10 (1.4%)
Specialty	Physician	19 (2.3%)
	Nurses	432 (53.0%)
	Allied health	315 (38.7%)
	Others	49 (6.0%)
Hospital name	Al Khor Hospital	4 (0.5%)
	Al Wakra Hospital	12 (1.4%)
	HMGH (Hazm)	295 (35.6%)
	Hamad General Hospital	482 (58.1%)
	PHCC	36 (4.3%)
Working with COVID-19 patient contact	Direct	574 (70.0%)
	In-direct	246 (30.0%)
How long you have been working in the designated COVID-19 facility?	1–3 months	60 (7.3%)
	4–6 months	75 (9.1%)
	7–12 months	51 (6.2%)
	>12 months	553 (66.9%)
	No experience	88 (10.6%)
	not mention	137 (16.6%)
Living status	Alone	624 (75.6%)
	With family	64 (7.8%)
	With others	222 (28.6%)
Family members	≤2	445 (57.3%)
	3–5	87 (11.2%)
	6–7	23 (3.0%)
	≥7	21 (2.5%)
Any family member/colleague/friend tested positive	I do not know	219 (26.4%)
	No	589 (71.0%)
	Yes	15 (1.9%)

In this sample, physicians made up 2.3% of the workforce, nurses (53%), allied healthcare workers including pharmacists, rehabilitation staff, dentists, dietitians, educators, researchers, technicians, respiratory therapists made up 38.7%, and other hospital staff such as administrative staff and engineers made up 6%. Most of the healthcare workers (70%) had direct contact with a COVID-19 positive patient and 67% were working in the COVID-19 facility last 12 months. About 76% of healthcare workers lived with their families, and 57.3% had three to five family members. 76% of healthcare workers who had the PCR test done at least four times or more had 71% of their relatives, co-workers or friends who tested positive.

**Figure 1:**
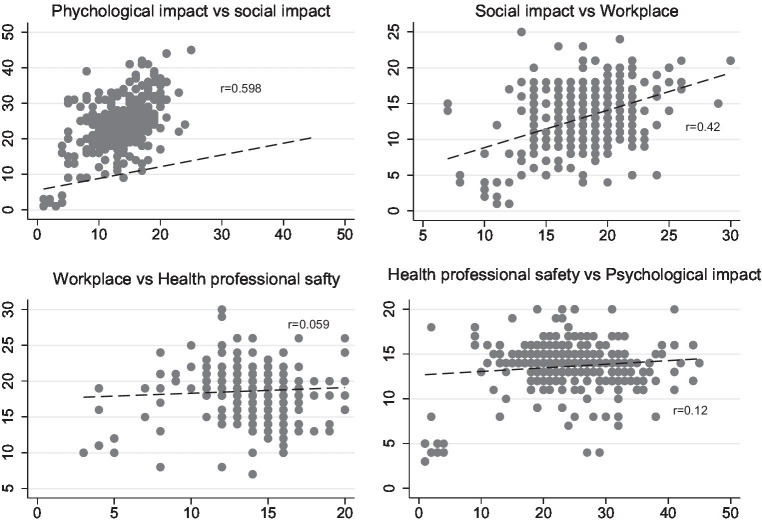
Correlation between psychosocial impact, social impact, workplace and health profession safety.

Figure 1 shows the correlation between all four variables, i.e., psychological, social, workplace and health professional safety.

The mean of psychological impact, social impact, workplace and health professional safety scores of the participants were 23.6 ± 7.37, 13.3 ± 4.0, 18.5 ± 3.3 and 13.6 ± 2.4, respectively.

Females had considerably greater (13.9 ± 1.7) health professional safety as compared to males (13.5 ± 2.7, *p* = 0.03), while males had a significantly larger (24.2 ± 8.3) psychological impact as compared to females (22.4 ± 4.9, *p* ≤ 0.001).

When compared to married and widowed/divorced individuals, those who were single had a considerably higher psychological and social impact (*p* = 0.004 and *p* = 0.022), respectively. Married people (18.6 ± 3.5), followed by single people (18.5 ± 2.9), had stronger workplace impacts than widowed or divorced people (14.9 ± 3.4), *p* = 0.013. As comparison to individuals who earned a bachelor’s, master’s, or diploma, healthcare practitioners who earned a Ph.D. had better psychological, social impact, and health professional safety (*p* < 0.001, *p* < 0.001 and *p* = 0.01), respectively. North Americans (30.2 ± 8.0) were more psychologically affected than Europeans (24.7 ± 13.5) and Asians (23.7 ± 8.0) in terms of nationality *p* = 0.035.

Compared to nurses, allied health professionals, physicians, and others had greater psychological, social, and occupational effects (*p* < 0.001), respectively.

Other factors like indirect exposure with COVID-19 patients, those are not having any experience with COVID-19 had significantly higher psychological and social impact *p* < 0.001, *p* < 0.001 and *p* < 0.001, *p* = 0.002, respectively. Those were living with others had greater psychological and social impact as compared to those who were with family and single *p* < 0.001 and *p* < 0.001, respectively.

In terms of family members those were having 6–7 family members and their family members, relatives and colleagues tested positive had significantly higher psychological impact *p* < 0.001 and *p* = 0.02 (Table 2).

**Table 2:** The association between socio demographic factors, psychological, social, workplace and health professional safety.

Variables	*N*	Psychological impact, mean ± SD	Social impact, mean ± SD	Workplace, mean ± SD	Health professional safety, mean ± SD
**Gender**					
Female	282	22.4 ± 4.9	13.5 ± 2.9	18.5 ± 2.8	13.9 ± 1.7
Male	544	24.2 ± 8.3	13.2 ± 4.5	18.5 ± 3.6	13.5 ± 2.7
*p*-value		<0.001	0.37	0.73	0.031
**Marital status**					
Married	587	23.3 ± 7.9	13.2 ± 4.3	18.6 ± 3.5	13.6 ± 2.6
Single	235	24.6 ± 5.5	13.6 ± 3.3	18.5 ± 2.9	13.7 ± 2.0
Widow/divorced	7	17.0 ± 9.6	9.4 ± 5.4	14.9 ± 3.4	11.6 ± 4.5
*p*-value		0.004	0.022	0.013	0.079
**Education level**					
Bachelor	627	23.4 ± 7.4	13.1 ± 4.1	18.6 ± 3.4	13.6 ± 2.4
Diploma	44	19.2 ± 5.4	12.2 ± 4.1	17.7 ± 2.5	13.4 ± 2.8
Master	115	25.7 ± 7.4	14.0 ± 3.6	18.4 ± 3.4	13.3 ± 2.5
Ph.D.	39	26.3 ± 5.6	15.5 ± 2.8	19.1 ± 2.1	14.8 ± 1.5
*p*-value		<0.001	<0.001	0.21	0.01
**Nationality**					
African	104	23.4 ± 4.7	13.3 ± 2.9	18.3 ± 3.1	13.3 ± 2.1
Asia	596	23.7 ± 7.3	13.4 ± 4.0	18.7 ± 3.3	13.8 ± 2.3
Europe	6	24.7 ± 13.5	14.3 ± 2.1	19.3 ± 0.8	14.2 ± 0.4
North America	10	30.2 ± 8.0	15.4 ± 5.9	18.0 ± 4.0	14.4 ± 0.8
*p*-value		0.035	0.39	0.65	0.13
**Profession**					
Physician	19	29.8 ± 5.6	16.4 ± 3.0	21.2 ± 2.3	12.4 ± 2.2
Nurses	432	22.5 ± 8.3	12.7 ± 4.4	18.7 ± 3.8	13.6 ± 2.8
Allied health	315	23.3 ± 7.3	13.2 ± 4.1	18.5 ± 3.3	13.6 ± 2.5
Others	49	26.7 ± 7.7	14.1 ± 3.9	18.3 ± 2.4	13.5 ± 2.4
*p*-value		<0.001	<0.001	<0.001	0.12
**Hospital**					
Al Khor Hospital	4	30.0 ± 1.2	14.5 ± 2.9	20.5 ± 0.6	14.5 ± 0.6
Al Wakra Hospital	12	29.1 ± 10.6	16.6 ± 5.1	16.6 ± 2.9	13.8 ± 0.4
HMGH (Hazm)	295	22.4 ± 9.5	12.1 ± 5.1	18.0 ± 4.2	13.7 ± 3.2
Hamad General Hospital	482	24.1 ± 5.5	13.9 ± 2.9	19.0 ± 2.5	13.6 ± 1.8
PHCC	36	25.6 ± 6.9	13.4 ± 4.4	17.1 ± 4.4	13.1 ± 2.7
*p*-value		<0.001	<0.001	<0.001	0.63
**Working with COVID-19 patient contact**					
Direct	574	22.9 ± 7.7	12.9 ± 4.2	18.5 ± 3.5	13.7 ± 2.6
In-direct	246	25.4 ± 6.3	14.1 ± 3.5	18.7 ± 2.8	13.3 ± 2.0
*p*-value		<0.001	<0.001	0.43	0.038
**How long you have been working in the designated COVID-19 facility?**					
1–3 months	60	22.8 ± 6.7	14.0 ± 3.9	17.5 ± 3.3	13.6 ± 1.9
4–6 months	75	25.6 ± 6.2	13.8 ± 3.8	18.8 ± 2.9	13.6 ± 1.9
7–12 months	51	22.8 ± 8.9	12.7 ± 4.5	18.8 ± 3.8	12.8 ± 3.6
>12 months	553	23.1 ± 7.4	13.0 ± 4.1	18.6 ± 3.3	13.8 ± 2.4
No experience	88	26.7 ± 6.3	14.7 ± 3.2	18.1 ± 3.6	12.9 ± 2.3
*p*-value		<0.001	0.002	0.076	0.005
**Living status**					
Alone	137	24.5 ± 7.2	13.2 ± 4.1	18.5 ± 3.0	13.7 ± 2.6
With family	624	23.2 ± 7.4	13.0 ± 4.0	18.6 ± 3.4	13.6 ± 2.5
With others	64	26.9 ± 6.8	16.3 ± 3.5	18.4 ± 2.7	13.3 ± 1.5
*p*-value		<0.001	<0.001	0.87	0.42
**Family numbers**					
≤2	222	23.8 ± 6.6	13.1 ± 3.4	18.9 ± 3.0	13.7 ± 2.1
3–5	445	23.0 ± 7.7	13.4 ± 4.1	18.5 ± 3.3	13.5 ± 2.5
6–7	87	26.7 ± 6.1	14.3 ± 3.9	18.3 ± 3.4	13.7 ± 2.3
≥7	23	24.7 ± 5.8	13.7 ± 4.9	18.5 ± 4.1	14.1 ± 1.9
*p*-value		<0.001	0.1	0.44	0.4
**Any family member/colleague/friend tested positive**					
I do not know	21	27.3 ± 4.9	14.9 ± 1.6	18.7 ± 4.0	12.7 ± 0.7
No	219	22.9 ± 8.8	12.5 ± 4.7	18.0 ± 3.2	13.6 ± 2.8
Yes	589	23.8 ± 6.8	13.5 ± 3.8	18.7 ± 3.3	13.6 ± 2.3
*p*-value		0.02	0.001	0.022	0.2

Table 3 shows the adjusted relationship between sociodemographic characteristics, workplace, social, and health professional safety. Age was significantly associated with psychological impact Coef. 0.25: 95% CI (0.16, 0.35). Men had more of an emotional influence Coef. 2.28: 95% CI (0.94, 3.61); *p* = 0.001 and lower health professional safety Coef. −0.49: 95% CI (−0.83, −0.12); *p* = 0.009 as compared to females.

**Table 3:** Multiple linear regression for psychological impact, social impact, workplace and health professional safety.

Variables	Psychological impact		Social impact		Workplace		Health professional safety	
	*β* (95% CI)	*p*-value	*β* (95% CI)	*p*-value	*β* (95% CI)	*p*-value	*β* (95% CI)	*p*-value
Age	0.25 (0.16, 0.35)	<0.001^*^	0.07 (0.01, 0.12)	0.013	0.07 (0.03, 0.11)	0	0.003 (−0.02, 0.03)	0.813
**Gender**								
Female	Ref						Ref	
Male	2.28 (0.94, 3.61)	0.001^*^	—	—	—	—	-0.49 (−0.85, −0.12)	0.009
**Marital status**								
Married	Ref		Ref		Ref			
Single	2.81 (1.07, 4.55)	0.002^*^	−0.25 (−1.19, 0.69)	0.6	0.56 (−0.05, 1.17)	0.07	—	—
Widow/divorced	1.51 (−7.6, 10.61)	0.745	−1.01 (−4.36, 2.35)	0.556	−4.06 (−6.44, −1.68)	0.001^*^	—	—
**Education level**								
Bachelor	Ref		Ref				Ref	
Diploma	−4.22 (−6.68, −1.75)	0.001^*^	0.08 (−1.25, 1.41)	0.909	—	—	−0.34 (−1.10, 0.43)	0.389
Master	0.84 (−0.87, 2.54)	0.336	0.65 (−0.27, 1.56)	0.165	—	—	−0.02 (−0.54, 0.51)	0.946
Ph.D.	−0.37 (−3.18, 2.44)	0.795	0.08 (−1.45, 1.6)	0.922	—	—	1.47 (0.59, 2.36)	0.001^*^
Nationality	Ref							
African								
Asia	1.11 (−0.52, 2.74)	0.181	—	—	—	—	—	—
Europe	−1.08 (−6.73, 4.58)	0.708	—	—	—	—	—	—
North America	4 (−0.92, 8.93)	0.111	—	—	—	—	—	—
**Specialty**								
Physician	Ref		Ref		Ref			
Nurses	−3.6 (−7.4, 0.19)	0.063	−2.03 (−4.03, −0.03)	0.047	−2.03 (−3.54, −0.53)	0.008	—	—
Allied health	−5.29 (−9.2, −1.38)	0.008	−2.3 (−4.35, −0.25)	0.028	−3.26 (−4.77, −1.75)	<0.001^*^	—	—
Others	−2.2 (−6.66, 2.27)	0.334	−2.38 (−4.72, −0.05)	0.045	−2.98 (−4.73, −1.23)	0.001^*^	—	—
**Hospital**								
Al Khor Hospital	Ref		Ref		Ref			
Al Wakra Hospital	−1.17 (−8.81, 6.48)	0.764	0.43 (−3.96, 4.81)	0.848	−4.72 (−8.34, −1.1)	0.011	—	—
HMGH (Hazm)	−6.03 (−12.93, 0.87)	0.087	−2.1 (−6.03, 1.83)	0.294	−3.19 (−6.37, −0.01)	0.049	—	—
Hamad General Hospital	−4.29 (−11.15, 2.57)	0.219	−1.12 (−5.02, 2.78)	0.573	−2.23 (−5.38, 0.92)	0.166	—	—
PHCC	−5.04 (−12.27, 2.19)	0.172	−2.2 (−6.28, 1.89)	0.291	−3.14 (−6.47, 0.2)	0.065	—	—
**Working with COVID-19 patient contact**								
Direct	Ref		Ref				Ref	
In-direct	0.63 (−0.85, 2.1)	0.405	1.03 (0.27, 1.78)	0.008	—	—	−0.14 (−0.56, 0.29)	0.531
**How long you have been working in the designated COVID-19 facility?**								
1–3 months	Ref		Ref				Ref	
4–6 months	2.19 (−0.54, 4.92)	0.116	−0.13 (−1.54, 1.28)	0.856	—	—	0.17 (−0.66, 0.99)	0.695
7–12 months	−1.2 (−4.06, 1.66)	0.411	−0.74 (−2.31, 0.83)	0.354	—	—	−0.65 (−1.56, 0.27)	0.165
>12 months	−0.21 (−2.45, 2.02)	0.851	−0.58 (−1.8, 0.63)	0.345	—	—	0.34 (−0.34, 1.03)	0.327
No experience	3.04 (0.3, 5.78)	0.03	0.17 (−1.29, 1.63)	0.821	—	—	−0.45 (−1.32, 0.43)	0.319
**Living status**								
Alone	Ref		Ref					
With family	0.74 (−1.32, 2.8)	0.481	−1.1 (−2.17, −0.04)	0.042	—	—	—	—
With others	0.92 (−1.97, 3.8)	0.533	1.69 (0.2, 3.17)	0.026	—	—	—	—
**Family numbers**								
≤2	Ref		Ref					
3–5	−0.55 (−1.83, 0.73)	0.402	−0.14 (−0.83, 0.55)	0.695	—	—	—	—
6–7	1.63 (−0.37, 3.62)	0.11	0.62 (−0.44, 1.68)	0.249	—	—	—	—
≥7	2.02 (−1.23, 5.26)	0.222	1.13 (−0.68, 2.93)	0.221	—	—	—	—
**Any family member/colleague/friend tested positive**								
I do not know	Ref		Ref		Ref			
No	1.69 (−3.41, 6.8)	0.515	−0.09 (−2.21, 2.03)	0.931	−2.14 (−3.73, −0.56)	0.008	—	—
Yes	1.79 (−3.21, 6.8)	0.482	1.03 (−1.02, 3.07)	0.324	−1.57 (−3.08, −0.06)	0.042	—	—

Model 1: psychological impact adjusted with age, gender, marital status, education level, nationality, specialty, hospital, working with COVID-19 patient, working experience, living status, family members, any family member colleague. Model 2: social impact adjusted with age, marital status, education level, specialty, hospital, working with COVID-19 patient, working experience, living status, family members, any family member colleague. Model 3: workplace adjusted with age, marital status, specialty, hospital, any family member colleague. Model 4: health professional safety adjusted with age, gender, education level, working with COVID-19 patient, working experience.

In comparison to married, being single had higher psychological impact Coef. 2.81 95% CI (1.07, 4.55); *p* = 0.002 and being widow/divorced had lower workplace impact Coef. −4.06: 95% CI (−6.44, −1.68); *p* = 0.001. When compared to those who earned a bachelor’s degree, those with a diploma had a significantly lower psychological effect Coef. −4.22: 95% CI (−6.68, −1.75); *p* = 0.001 and those who completed a Ph.D. had higher health professional safety Coef. 1.47: 95% CI (0.59, 2.37), *p* = 0.001.

The psychological effect was lower among the allied health professionals Coef. −5.29: 95% CI (−9.2, −1.38); *p* = 0.008; nurses Coef. −2.03: 95% CI (−4.03, −0.03); *p* = 0.047; allied health professionals Coef. −2.3: 95% CI (−4.35, −0.25); *p* = 0.028; and others Coef. −2.38: 95% CI (−4.72, −0.05); *p* = 0.045 had lower social impact as compared to physicians. As compared to physicians, nurses Coef. −2.03: 95% CI (−3.54, −0.53), *p* = 0.008, allied health professional Coef. −3.26: 95% CI (−4.77, −1.75), *p* = <0.001 and others Coef. −2.98: 95% CI (−4.73, −1.23), *p* = 0.001 had lower workplace impact. Alwakra hospital also had lower workplace impact Coef. −4.72 95% CI (−8.34, −1.1), *p* = 0.011 as compared to Alkhor hospital.

Those who worked with COVID-19 patients indirectly had greater social impacts Coef. 1.03 95% CI (0.27, 1.78), *p* = 0.008 compared to those working directly with COVID-19 patients. Health workers who lived with families had a lower social impact on Coef. −1.20: 95% CI (−2.17, −0.04), *p* = 0.042, and those living with others had a higher social impact Coef. 1.70: 95% CI (0.2, 3.17), *p* = 0.026 compared to those living alone.

Those who know that their family members, co-workers or friends tested positive had a lower social impact Coef. −1.57: 95% CI (−3.08, −0.06), *p* = 0.042, and those who have no friends, colleagues, or family members had a lower social impact Coef. −2.14: 95% CI (−3.73, −0.56), *p* = 0.008 compared to those who were unaware that their friends, co-workers and family members have tested positive.

## 4. Discussion

As previously mentioned, numerous reports have detailed the various coping strategies adopted by healthcare professionals. These approaches include seeking psychological support through counseling and therapy, engaging in stress-reduction activities like physical exercise, meditation, and yoga, fostering peer support from family and friends, and prioritizing effective self-care routines, among others. These efforts played a pivotal role in maintaining resilience and upholding an exceptional standard of patient care during this challenging period (27–30). Additionally, some scholars have highlighted individual and environmental factors, such as incidents of violence or psychiatric illnesses, prolonged wait times, understaffing in emergency rooms, a history of drug or alcohol abuse, and unrestricted public movement, as contributing to the challenges faced in healthcare settings (34).

This study aimed to elucidate the psychosocial experiences of HCWs who worked undergone the COVID-19 crisis. Drawing from the accounts of the participants, various domains were explored, including the psychological impact, social consequences, safety, and workplace.

### 4.1. Levels of anxiety, depression, and insomnia among Qatar HCWs

In this study, healthcare workers (HCWs) experienced moderate psychological distress, which aligns with international research showing high levels of anxiety and depression among HCWs during the COVID-19 pandemic. The unprecedented challenges posed by the pandemic, such as increased work demands, fear of infection, and concerns about transmitting the virus, contribute to the psychological burden on HCWs (5, 35–43).

The social impact observed among HCWs in Qatar indicates a moderate disruption to social connections, consistent with international studies highlighting social isolation and loneliness experienced by HCWs (44–48). Implementing physical distancing measures and reducing social interactions have contributed to a sense of isolation among HCWs (48).

The findings indicate a high perception of workplace safety among participants, reflecting their confidence in the safety measures implemented by healthcare institutions in Qatar. This finding confirms that healthcare institutions have prioritized the safety of HCWs in the region. Studies conducted in Taiwan (49) and Saudi Arabia (50) have emphasized the importance of personal protective equipment (PPE) and infection control measures in reducing the risk of COVID-19 transmission among HCWs. Adequate provision of PPE, adherence to infection control guidelines, regular testing protocols, and vaccination have contributed to the high safety perception among participants.

Regular testing programs in Qatar enable early detection of COVID-19 cases among HCWs, facilitating prompt isolation and reducing the risk of transmission within healthcare settings (50, 51). Vaccination is another crucial factor in ensuring the safety of HCWs, and Qatar has made significant efforts to provide COVID-19 vaccines to its healthcare workforce (42, 52). Prioritizing immunization helps minimize the risk of infection and associated complications. This approach aligns with international best practices, as studies conducted in countries like the United States (53) and Canada (54) have also emphasized the importance of regular testing and vaccination in protecting HCWs from COVID-19.

These findings support the global understanding of the benefits of vaccination and regular testing as essential measures for safeguarding the health and safety of HCWs.

### 4.2. Factors influencing psychological distress in Qatar

Our findings imply that age significantly impacts how the pandemic affects HCWs psychologically. Older HCWs may have heightened concerns about their vulnerability to the virus due to age-related health conditions, leading to increased psychological distress (55). Additionally, their professional experience and knowledge may contribute to higher levels of responsibility and pressure, further impacting their psychological well-being (56). International studies have also reported similar associations between age and psychological impact among HCWs. For instance, a study conducted in Jordan found higher levels of psychological distress among older HCWs compared to younger individuals (57). Conversely, a study in Saudi Arabia indicated that younger HCWs experienced more psychological distress (58). These findings underscore the importance of considering age-related factors when addressing the psychological well-being of HCWs during the COVID-19 pandemic.

Our findings indicate that females perceived higher levels of safety, while males reported a more significant psychological impact. The higher perception of safety among females may be attributed to their greater compliance with infection control measures and adherence to safety protocols (59). Additionally, female HCWs may possess a heightened awareness of COVID-19 risks and a stronger sense of responsibility toward their safety and that of their colleagues. On the other hand, males may experience additional stress and emotional burden due to societal expectations related to strength, resilience, and leadership in their professional roles (60). These unique challenges male HCWs face may contribute to their higher reported psychological impact. Therefore, it is crucial to consider these gender-specific factors when addressing the well-being of HCWs during the pandemic.

Furthermore, the results of this study are consistent with those of a study by Alhofaian et al. (61) carried out in Saudi Arabia, which also revealed that female HCWs perceived higher levels of safety than males. This suggests that gender differences in safety perception may transcend the specific context of this study. However, studies conducted in other regions, such as the US (53), have yielded different results, indicating that gender differences in safety perception may vary across cultural, social, and organizational contexts. Therefore, it is essential to consider these contextual factors when interpreting and generalizing the findings of this study.

The study findings revealed significant associations between marital status, educational level, and nationality, and the psychological, social, workplace, and health professional safety impacts experienced by HCWs during the pandemic. For example, single individuals reported higher psychological and social impacts than married and widowed/divorced individuals (62, 63). Married individuals, on the other hand, reported more substantial workplace impacts compared to widowed or divorced individuals (64). These findings suggest that marital status can influence the experiences and challenges faced by HCWs during the pandemic. In addition, the study findings indicate that HCWs with a Ph.D. qualification demonstrated better psychological and social impact and health professional safety than those with lower educational levels (65). This suggests that higher levels of education contribute to better coping strategies and a greater sense of control among HCWs.

On the other hand, the study discovered that North Americans suffered more significant psychological effects than Europeans and Asians, possibly due to cultural variations, healthcare systems, and the severity of the pandemic in various regions. When comparing the findings of this study with international, Middle Eastern, and Arabic studies, several similarities and differences emerge. For instance, Tan’s et al. (66) study in Singapore found similar results regarding the higher psychological impact single HCWs experience. This suggests that the association between marital status and psychological impact extends beyond regional boundaries. Additionally, studies conducted in Turkey (67) and India (68) showed that higher educational qualifications are associated with better psychological well-being and coping mechanisms among HCWs, aligning with the findings of this study.

However, limited research comparing North Americans, Europeans, and Asians regarding psychological impact among HCWs during the pandemic was found, making this finding regarding nationality a novel contribution that warrants further investigation.

Physicians in this study experienced more significant psychological, social, and occupational impacts than nurses, allied health professionals, and others. The higher psychological impact among physicians can be attributed to their direct involvement in diagnosing and treating COVID-19 patients, which exposes them to higher stress levels and emotional burdens. Difficult decisions regarding patient care, resource allocation, and ethical dilemmas further contribute to their psychological distress. The demanding nature of their profession, long working hours, and limited social engagement outside of work also play a role. Similar patterns have been observed in studies conducted in the United States (69) and Belgium (70), highlighting the global nature of physicians’ challenges. These findings underscore the importance of targeted interventions to support physicians’ well-being.

Participants without any prior experience with COVID-19 had significantly higher psychological and social impacts than those with previous exposure (71). This can be attributed to limited knowledge and understanding of the virus, increased anxiety, and uncertainty. The fear of contracting the virus and its potential consequences for personal and loved ones’ health further contribute to the observed impact. Moreover, individuals living with others, such as roommates or colleagues, experienced more significant psychological and social impacts than those living with their families or being single (72). This suggests that the dynamics of shared living spaces and interactions with others may contribute to increased stress and emotional burden. The challenges of maintaining physical distance, addressing potential conflicts, and navigating shared spaces could all contribute to the observed impact.

While these findings provide valuable insights, comparing them with international, Middle Eastern and Arabic studies is challenging due to the novelty of this specific discovery. Therefore, this finding represents a novel discovery and highlights the need for further research to understand the underlying mechanisms and explore potential interventions.

## 5. Limitations

In spite of the findings presented in this study, it is important to acknowledge several limitations. The first limitation of this study is that the measurements, they were conducted after a peak of COVID-19. This timing may have influenced the psychosocial working conditions experienced during the data collection period. It is worth considering that the results might have varied if the measurements had been taken during peak hospitalization periods. The second limitation is that only participants who had given permission in 2019 were contacted to participate. This approach introduces the possibility of selection bias, as the sample may not accurately represent the entire population of interest.

## 6. Implications for the healthcare sector in Qatar and beyond

The findings of this study have several implications for the healthcare sector in Qatar and beyond. First, acknowledging the psychological distress that HCWs experience emphasizes the need for extensive mental health support services and interventions. Therefore, healthcare organizations in Qatar should prioritize providing resources, such as access to mental health services and tailored coping mechanisms, to address the specific needs of HCWs. Additionally, efforts should be made to foster supportive environments, promote peer support programs, and facilitate opportunities for HCWs to maintain social connections while adhering to safety protocols.

The study’s findings regarding the perceived safety of HCWs highlight the effectiveness of infection control measures, PPE, regular testing, and vaccination in protecting HCWs. These measures should continue to be implemented and prioritized in Qatar’s healthcare institutions to ensure the safety of HCWs. Furthermore, these findings are consistent with international best practices, emphasizing the importance of regular testing and vaccination in protecting HCWs from COVID-19. Qatar’s adherence to these practices aligns with global recommendations and demonstrates its commitment to the safety and well-being of its healthcare workforce.

## 7. Conclusion

In conclusion, this study examined anxiety, depression, insomnia, psychological impact, social impact, workplace safety, and health professional safety among HCWs in Qatar during the COVID-19 pandemic. The findings revealed moderate levels of psychological distress, disruption to social connections, and perceived safety among HCWs. In addition, age, gender, marital status, educational level, nationality, and designation were identified as significant factors influencing the psychological and social impacts experienced by HCWs. The study also highlighted the importance of robust infection control measures, adequate PPE, regular testing, and vaccination in ensuring the safety and well-being of HCWs.

## Data availability statement

The raw data supporting the conclusions of this article will be made available by the corresponding author upon request.

## Ethics statement

The studies involving humans were approved by Medical Research Center (MRC)/Hamad Medical Corporation/Qatar. The studies were conducted in accordance with the local legislation and institutional requirements. Written informed consent for participation in this study was provided by the participants’ legal guardians/next of kin.

## Author contributions

AA-Q: Conceptualization, Investigation, Methodology, Supervision, Writing – original draft, Writing – review & editing. KS: Formal analysis, Writing – review & editing. EM: Writing – review & editing. AN: Writing – review & editing. RA-Z: Writing – review & editing. AY: Supervision, Writing – review & editing. OA: Supervision, Writing – review & editing. AA-A: Supervision, Writing – review & editing.
